# Supplementary material for: Atomic Force Microscopy Micro-Indentation Methods for Determining the Elastic Modulus of Murine Articular Cartilage
Source: Sensors (Basel). 2023 Feb 7;23(4):1835. doi: 10.3390/s23041835 (PMC9967621; doi:10.3390/s23041835)
Supplement: Supplementary file 1 [file sensors-23-01835-s001.zip › sensors-2133449-supplementary.pdf]

SUPPLEMENTAL FILES (FIGURES, TABLES AND VIDEO LEGEND)

Supplementary Figures

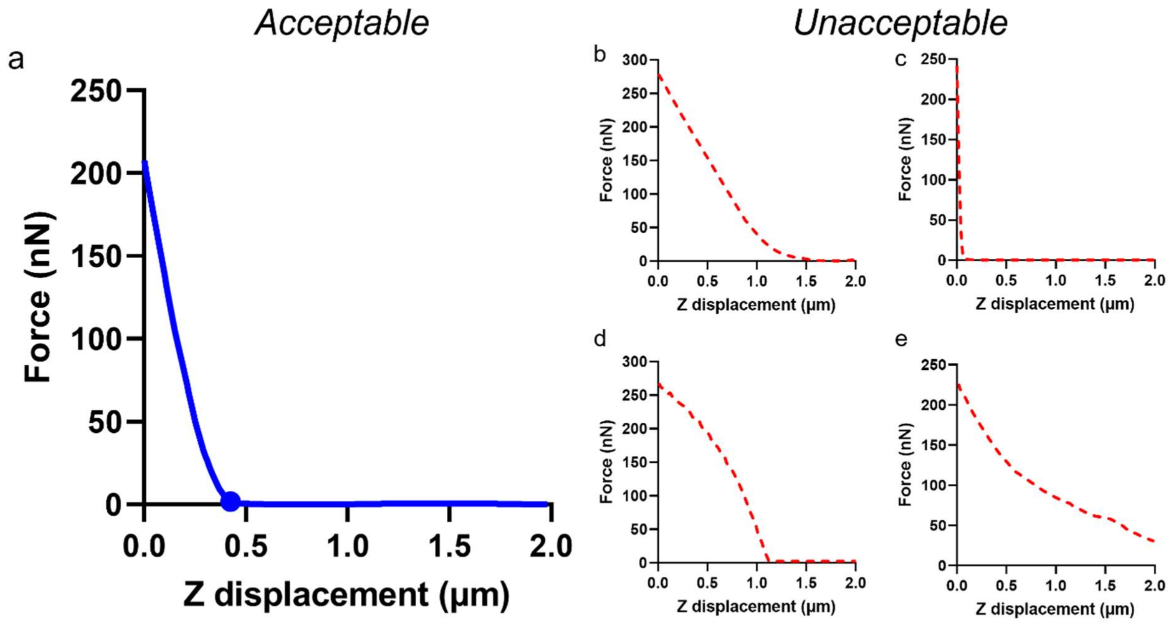

Figure S1: Examples of acceptable (a, blue solid) and unacceptable indentation curves (b-e, red dashed). Reasons to discard a curve include a dramatically different appearance, indicating indentation of a structure other than articular cartilage, for example a cell (b) or adjacent bone (c), an inverted curve (d), or lack of 'zero' data (e).

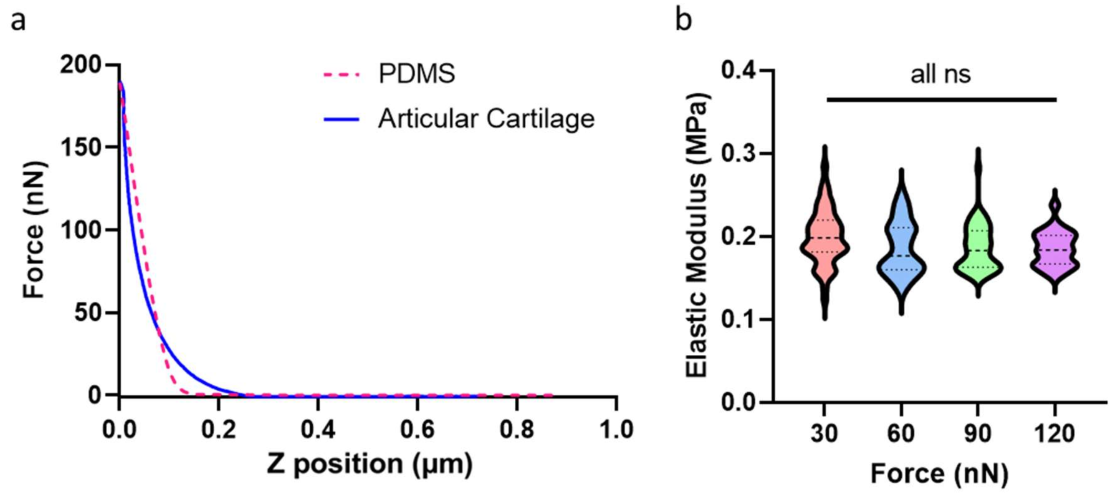

Figure S2: Comparison of curves generated from the indentation of a PDMS gel, articular cartilage, or tape. (a) PDMS gel (red dash) and articular cartilage (blue solid). For both indentations, the same experimental parameters were used. Aside from the differences in the gradient of the curves (due to the different elastic moduli) in the linear region, the lower portions of the curve represent the differences between a gel and a biological sample. Where the PDMS has a relatively sharp contact point, the cartilage sample has a gentle curve from the contact point until the linear region. (b) Elastic modulus measurements made on tape adjacent to sample. Tape was approximately 10 times softer than cartilage and there was no effect with indentation force.

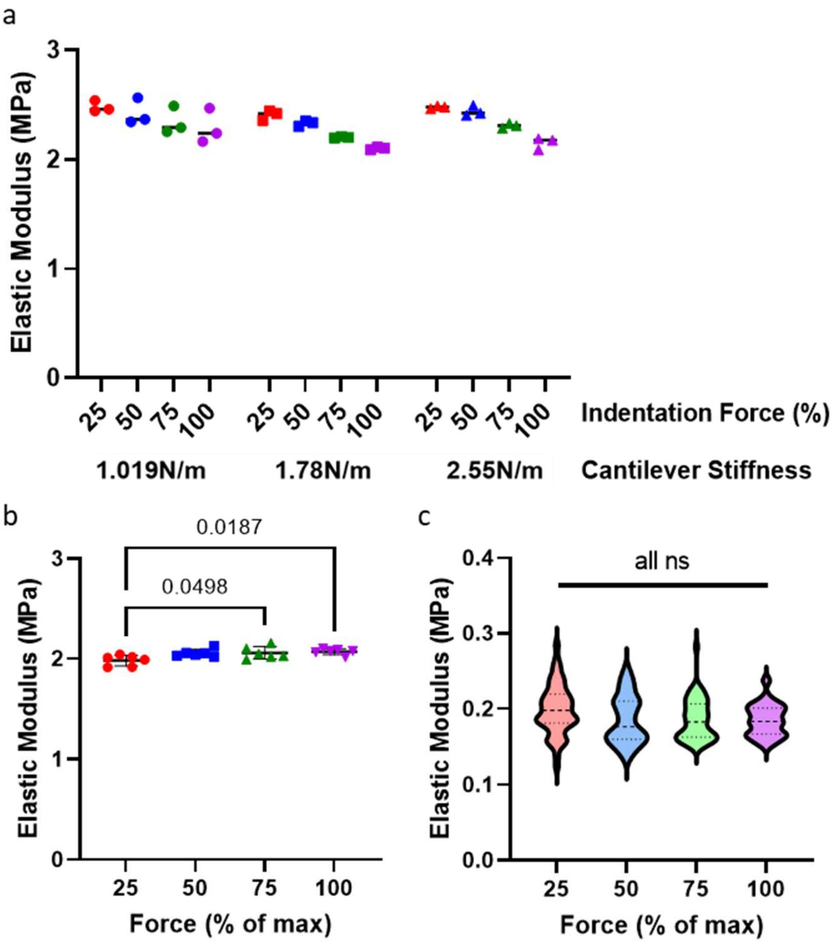

Figure S3: Indentation force of PDMS gel, cartilage, and tape as a percentage of maximum force applied. (a) Group of 3 individual tips with listed cantilever stiffness k = 1N/m, showing effect of indentation force on PDMS gel. Actual cantilever stiffnesses are listed below. All tips were comparable and showed a significant decrease in modulus with increased indentation force. (25 vs 50% $p = 0.0002$, 25 vs 100% $p < 0.0001$). (b) Effect of indentation force on measured elastic modulus of articular cartilage, presented as a percentage of maximum force appliable. (c) Elastic modulus measurements made on tape adjacent to sample. Tape was approximately 10 times softer than cartilage and there was no effect with indentation force, presented as a percentage of maximum force appliable.

Supplementary Tables

Table S1: Ranges of experimental AFM parameters tested.

Variable	Range
Cantilever stiffness	0.08-20 N/m
Indentation force	15-3400 nN
Ramp rate	0.5-1.5 Hz
Ramp size	1-10 μm

Table S2: Force limits for cantilevers as determined by thermal tune and detector limits

Cantilever stiffness k (N/m)	Nominal stiffness k (N/m)	Force limit (nN)
0.089	0.06	22
0.094	0.06	24
1.019	1	120
1.78	1	380
2.55	1	450
5.67	8.9	1500
11.65	8.9	2100
17.97	16	3000
20.4	16	3700

Table S3: Specifications for silicon cantilevers (Bruker), specifically for those with nominal stiffess of 1N/m.

	Nominal value	Minimum	Maximum
Spring Constant k (N/m)	1	0.1	4.6
Length (µm)	110	105	115
Width (µm)	32.5	29.5	35.5
Thickness (µm)	1	0.5	1.5

Cryofilm sectioning
for AFM.mp4

Supplemental Video: Video of tape-based sectioning method for AFM sample preparation to complement photos in Figure 1
